# Mental Health Problems as a Risk Factor for Workplace Bullying: The Protective Effect of a Well-Functioning Organization

**DOI:** 10.1093/annweh/wxab040

**Published:** 2021-06-19

**Authors:** Michael Rosander

**Affiliations:** Department of Behavioural Sciences and Learning, Linköping University, Campus Valla, IBL, 581 83 Linköping, Sweden

**Keywords:** longitudinal data, mental health, probability sample, role clarity, sickness presenteeism, work environment hypothesis, workplace bullying

## Abstract

This study examined a strain–stressor association, *when* mental health problems may lead to subsequent workplace bullying, and a mechanism of *how* this can happen. I hypothesized that the association between mental health problems and bullying depends on the perceived role clarity and order in the organization, and that sickness presenteeism (SP) mediates this association. The study is based on a longitudinal probability sample drawn from the total number of employees in Sweden. Workplace bullying, mental health, SP, and role clarity and order in the organization were assessed using a questionnaire. The results showed that mental health problems are associated with an increased risk for subsequent bullying, consistent with previous findings. However, this risk depends on the level of role clarity and order in the organization. The results also show a partial indirect effect via SP. This means that if one has mental health problems and persists in coming to work although one should have stayed at home, the risk of bullying increases. The indirect effect depends also on the level of order in the organization. The findings suggest that individual deficits, such as mental health problems, are associated with subsequent bullying only if organizational deficits are also present.

**What’s important about this paper**

This study investigated when mental health problems may lead to subsequent workplace bullying, and a mechanism of how this can happen. The study extends knowledge in the field by adding the organizational level, where previous research has focussed only on personal or interpersonal aspects of mental health problems. The results showed that the risks associated with mental health problems depend on the level of role clarity and order in the organization, and that sickness presenteeism mediates this association. For both these aspects, this study contributes to knowledge by showing that individual deficits, such as having mental health problems, are associated with subsequent bullying only if organizational deficits are also present.

## Introduction

There is a wide agreement that workplace bullying can lead to mental health problems for the exposed person (e.g. Nielsen and Einarsen, 2012; Nielsen *et al.*, 2014; Einarsen and Nielsen, 2015). There is evidence also for a ‘reversed effect’, in which poor mental health can lead to exposure to bullying. Based on a systematic review and a meta-analysis, Nielsen *et al.* (2014) found an almost doubled risk for a longitudinal association between mental health problems and bullying. Mainly individual and interpersonal explanations have been proposed for the reversed effect, such as problems living up to expectations, a more negative view of the work environment, and a heightened sensitivity to behaviours interpreted as aggression (Nielsen and Einarsen, 2018). Research on antecedents or risk factors for workplace bullying in general has focussed since the 1990s mainly on different aspects of the work environment, and today there is a strong empirical support for the so called ‘work environment hypothesis’ (Salin and Hoel, 2020). The essence of this perspective was elegantly summed up as: ‘bullying seems to thrive where employees perceive contradictory expectations, demands and values in their job and where expectations are perceived as unclear or unpredictable’ (Salin and Hoel, 2020, p. 307). Workplace bullying is defined as a systematic and prolonged exposure to negative behaviours at work that the exposed have difficulties to protect themselves from (Einarsen *et al.*, 2020). Negative behaviours can be direct, such as verbally attacking or humiliating the target, and they can be indirect, such as spreading rumours, or simply ignoring or excluding the target. Bullying behaviours can also be person related, such as insults about the target’s private life or background, and they can be work related, such as withholding important information, or forcing the target to do trivial or unpleasant tasks. The current study contributes with new knowledge about how the individual and interpersonal aspects of mental health problems related to workplace bullying can be better understood in the context of the work environment hypothesis. The study addresses *when* mental health problems can lead to bullying, and it identifies a mechanism of *how* mental health problems can lead to bullying.

### Mental health problems and workplace bullying

Mental health problems and bullying at work are quite common. In Sweden, where the current study was conducted, it has been estimated that 15% of employees have some form of impaired mental health. About 5% reported severe anxiety and 3% had a depression diagnosis (Public Health Agency of Sweden, 2018). As for the prevalence of workplace bullying Rosander and Blomberg (2019) showed that 19% of employees were exposed to bullying behaviours to a degree that affected their health and well-being negatively—of those, 7% were exposed to ongoing bullying or severe bullying. Most studies on workplace bullying that have included measures of mental health problems, such as anxiety or depression, have focussed on the consequences of bullying, but some have included mental health problems as an antecedent or risk factor (e.g. Kivimaki *et al.*, 2003; Reknes *et al.*, 2014). Nielsen *et al.* (2014) found seven studies (with more than 18 000 participants), and a total odds ratio (OR) of 1.74 [95% confidence interval (CI) 1.44–2.12] for a longitudinal association between mental health problems and subsequent bullying. In an early study, Kivimaki *et al.* (2003) measured depression in a true prospective design, and estimated the risk for new victims at follow-up (OR 2.46). Finne *et al.* (2011) found that mental distress at baseline was associated with an increased risk of being bullied at follow-up 2 years later (OR 2.30). Nielsen *et al.* (2012) found an OR for exposure to bullying behaviours at follow-up of 2.5 if the target had baseline psychological distress, and almost the same increase for self-labelled victimization. They discussed the possibility that a poorly organized work environment leads to frustration and interpersonal conflicts prior to baseline, which could have created mental health problems, a stressor–strain relationship, and that these problems lead to bullying at follow-up, the strain–stressor relationship. This is an example of the possible reciprocal nature of mental health problems and bullying—that they contribute to a vicious circle (Nielsen *et al.*, 2014). The strain–stressor–strain process of mental health problems was discussed also by Zapf *et al.* (1996), and studied by Reknes *et al.* (2014), who found support for a vicious circle. The idea that mental health problems and bullying have a self-reinforcing relationship has, however, been questioned. In a 5-year prospective study, Einarsen and Nielsen (2015) showed that the presence of anxiety symptoms at baseline was associated with subsequent exposure to bullying behaviours, but only for men. Rosander *et al.* (2020) also showed that the association between mental health problems and subsequent bullying differs between men and women: only men experienced a reversed effect for self-labelled victimization.

### Theoretical perspectives on why mental health problems may lead to bullying

From a social interactionist perspective (Felson, 1992), aggression can be triggered by perceived rule violations. A violation may be that a person is perceived not to perform as expected, or a violation of the rules of social interaction. Failure to adhere to the expectations of a polite exchange and mutual support may be perceived as aggressive, or at least inappropriate, behaviour and may trigger an aggressive response. Aquino and Thau (2009) argued that employees who have high levels of negative affect, such as anxiousness or depression, may be perceived as demanding or difficult to deal with, and are thus more likely to become targets of aggression. This is similar to what Zapf *et al.* (1996) called the true strain–stressor hypothesis. The hypothesis states that people with mental health problems tend to view their environment more negatively, which may contribute to a more negative group climate. This may in turn create or intensify conflicts within the group, and create more social stressors. The tendency to view one’s environment more negatively may also have a direct impact on the interpretation of things happening in the organization, the ‘gloomy perception’ (de Lange *et al.*, 2005). In the interaction with others, the gloomy perception may make a person with mental health problems more sensitive to behaviours, and more prone to interpret them as negative. With a lower tolerance for behaviours construed as aggression, the threshold for interpreting behaviours as bullying may be lower (Nielsen and Einarsen, 2018). If an employee with mental health problems experiences more social stressors, the mental health problems might increase, creating a vicious circle (Zapf *et al.*, 1996).

### Mental health problems and sickness presenteeism

There is an association between mental health problems and sickness presenteeism (SP) (Burton *et al.*, 2004; McTernan *et al.*, 2013). McTernan *et al.* (2013) discussed the connection between the stigma of mental illness and showing up at work to prevent one’s condition becoming known. The consequences of SP for a person with mental health problems may be cognitive deficits (such as problems concentrating and difficulty in managing time), interpersonal problems, and a failure to achieve expected output (Burton *et al.*, 2004). Neto *et al.* (2017) found a strong negative association between loss of psychological well-being and the ability to concentrate at work. They also found an association between workplace bullying and the ability to complete one’s work—an indirect measurement of presenteeism. Conway *et al.* (2016) studied SP as a consequence of workplace bullying, and discussed the possibility that presenteeism is a risk factor for bullying. There are a few other studies on the association between bullying and presenteeism, for example, Ariza-Montes *et al.* (2021), who showed an association, but only for work-related bullying. Notelaers *et al.* (2018) showed that targets of severe bullying had high levels of presenteeism. Both studies used cross-sectional data and focussed on presenteeism as an outcome. However, as cross-sectional studies, they give information of association but not direction of the effect, so based on the previous studies there are results pointing to an association between presenteeism and bullying.

### Mental health problems and the work environment hypothesis

There is a wide agreement that the work environment has an important role in understanding why bullying occurs, the work environment hypothesis (Leymann, 1996; Einarsen, 2000; Salin and Hoel, 2020). The hypothesis states that organizational factors such as role ambiguity and role conflict are important antecedents of workplace bullying (Salin and Hoel, 2020). This line of reasoning has been in focus for a long time, but the effects of mental health problems have generally been regarded as a separate issue, not related to the work environment hypothesis. No previous studies have investigated possible moderators or mediators of the reversed association between mental health problems and workplace bullying.

### Hypotheses

In this study, I combine the organizational perspective from the work environment hypothesis with the individual or interpersonal perspective that focusses on what mental health problems may lead to in terms of subsequent workplace bullying (18-month time lag). First, a hypothesis based on the theoretical perspectives on why mental health problems may lead to bullying presented above as well as results from previous studies to establish that a reversed effect can be found:

*H1. Mental health problems at baseline are associated with subsequent exposure to workplace bullying.*

The second hypothesis includes the work environment hypothesis. I examine whether the work environment hypothesis is relevant also for people with mental health problems as described above, or whether the gloomy perspective mechanism and similar individual or interpersonal theoretical explanations take overhand and affect the occurrence of bullying, regardless of the work environment.

*H2. The association between mental health problems at baseline and subsequent workplace bullying depends on the perceived role clarity and order in the organization.*

Finally, a third hypothesis looks at a possible mechanism of the reversed effect. Previous studies, as described above, have shown an association between SP and cognitive and interpersonal problems. The stigma associated with mental health problems may make employees who experience these problems attempt to conceal them by showing up at work although they should have stayed at home. I propose that SP mediates the association between mental health problems and subsequent bullying, while the work environment hypothesis also plays a part.

*H3. The association between mental health problems at baseline and subsequent workplace bullying is mediated by the number of days of sickness presenteeism, and both the direct and indirect effect depend on the perceived role clarity and order in the organization.*

## Methods

### Design and participants

The study is based on a probability sample drawn by Statistics Sweden (scb.se/en) from the total number of employees in Sweden 18–65 years old, working at workplaces with 10 or more employees (about 3.3 million people). The baseline data were collected in the autumn of 2017 (*n* = 1854) and the follow-up data in the spring of 2019 (*n* = 1095). Only those who responded to the baseline questionnaire were invited to complete the follow-up. Statistics Sweden handled the distribution of questionnaires via mail to the participants’ home addresses. They added register data from the Swedish population register before sending the data to me. This meant that I never had access to any direct personal information, such as names or addresses, to ensure good research ethics. The project was approved by the Regional Ethical Review Board at Linköping University. Protocol number: #2017/336-32.

Of those who answered the questionnaire at both times, 174 had changed jobs during the 18 months between data collections. I excluded them from further analysis, as changing jobs will result in a new working environment, which makes it hard to separate the effects of the change of workplace from any continued influence from the same workplace.

The mean age of the participants was 50.1 years [standard deviation (SD) = 9.8]. They had worked at their current workplace on average 14.2 years (SD = 11.8). There were 58% women in the sample, 90% were born in Sweden, 55% were married, 14% had a managerial position, and 97% had a fixed contract. The majority (59%) had some form of university or college education, 1% had fewer than 9 years of schooling, 4% had only 9–10 years (compulsory school), and 35% had 11–12 years.

### Measures

Mental health problems were measured using the Hospital Anxiety and Depression Scale (HADS, Zigmond and Snaith, 1983). The HADS contains 14 items that cover anxiety and depression symptoms, each on a response scale with four alternatives with a score range from 0 to 3. An example of the items is, ‘I feel cheerful’ with possible responses from *not at all* to *most of the time*. The mean of the 14 items was used. The internal consistency (Cronbach’s alpha) was 0.90 at T1.

Workplace bullying was measured using both a behavioural experience method, and self-labelling based on a definition. The Negative Acts Questionnaire—Revised (NAQ-R, Einarsen *et al.*, 2009) contains 22 negative acts that cover work-related, person-related and physically intimidating behaviours. The NAQ-R has a frequency scale (*never*, *now and then*, *monthly*, *weekly*, *daily*), and asks for the participant’s exposure during the preceding 6 months. The Swedish version of the NAQ-R (Rosander and Blomberg, 2018) was used in this study. The internal consistency (Cronbach’s alpha) for the NAQ-R was 0.89 at T1, and 0.91 at T2. I used mean scores for the whole scale (score range 1–5) as well as cut-off scores for the sum of all 22 items (score range 22–110). Notelaers and Einarsen (2013) proposed cut-off scores at 33 and 45. They called a sum score in the range 33–44 ‘occasional bullying’, and one of 45 or higher ‘victim of bullying’. I also measured workplace bullying using self-labelling, based on a definition of bullying. The use of these two types of measure is recommended, as they capture slightly different aspects of the phenomenon (Nielsen *et al.*, 2020; Rosander *et al.*, 2020). The definition used included that bullying is a systematic and enduring negative treatment that is hard to defend against, followed by a question about having been exposed to that treatment during the preceding 6 months. The same frequency scale as that used in the NAQ-R (score range 1–5) was used.

Role clarity and order in the organization was measured using a scale from the Psychosocial Work Environment Questionnaire (PSYWEQ, Rosander and Blomberg, 2018) called ‘Roles in the organization’ (RIM, Rosander and Blomberg, 2019; Nielsen *et al.*, 2021). It contains six items that focus on: (i) unclear roles, responsibilities, and tasks; (ii) a clear division of tasks; (iii) clear roles; (iv) an orderly organization; (v) well-functioning routines and organization; and (vi) clear role expectations. The response scale for RIM is a seven-point Likert scale. Cronbach’s alpha at T2 was 0.89. High values mean clear roles and an orderly organization. The score range for the RIM is 1–7.

SP was estimated using a question from the PSYWEQ: ‘How many days have you been at work even though you should not have been working due to illness (in the last 12 months)?’ To give an idea of the extent of SP in the current sample, the mean number of days of SP was 3.59 (SD = 9.55). The variable was transformed to days per week in order to better correspond to the other study variables.

Covariates in the study were sex and age. Sex was added because there was a significant difference in mental health at baseline between men (HADS men = 0.58, SD = 0.46) and women (HADS women = 0.64, SD = 0.46), *t*(915) = −2.14, *P* = 0.033. There was also a small but significant difference for the RIM at follow-up between men (RIM men = 5.18, SD = 1.24) and women (RIM women = 5.35, SD = 1.20), *t*(918) = −2.08, *P* = 0.037. For age, there was a small but significant negative zero-order correlation for mental health and bullying (*r*-values between −0.12 and −0.15, *P*-values <0.001), and a positive correlation for RIM (*r* = 0.13, *P* < 0.001). I also adjusted for baseline bullying in all analyses.

### Attrition analyses

In the attrition analyses, I compared baseline demographic data and study variables for dropouts after the first data collection (T1) with those who participated in the follow-up. There were no significant differences in the proportions of men and women, but the dropouts were younger, 46.6 years (SD = 11.8), compared with 50.1 years (SD = 9.8), *t*(1677) = −6.48, *P* < 0.001. The dropouts had been somewhat more bullied (difference in mean NAQ-R = 0.05, *t*(1671) = 2.96, *P* = 0.003), and had more severe mental health problems (difference in mean HADS = 0.08, *t*(1670) = 3.49, *P* < 0.001). The differences in the incidence of bullying and severity of mental health problems were not large, however, previous research has shown that there is a greater risk of expulsion from the labour market after exposure to workplace bullying (Glambek *et al.*, 2015). It is possible that some of those who dropped out no longer had a job, and could not respond to the follow-up questionnaire, so the differences are not surprising. Dropouts viewed the order and clarity of their organization differently. The mean RIM for dropouts was 5.04 (SD = 1.36) and for those filling in the questionnaire at both times 5.22 (SD = 1.25), *t*(1675) = −2.78, *P* = 0.005.

### Statistical analyses

IBM SPSS, version 27 for the Mac, was used for all statistical analyses. I used logistic regression to test H1, and investigated the association between mental health problems (mean HADS at T1) and workplace bullying at T2, using the cut-off at 33 for the NAQ-R, as suggested by Notelaers and Einarsen (2013). For H2, I conducted a moderation analysis using model 1 in Hayes’ PROCESS macro, version 3.5 (Hayes, 2018). I investigated whether role clarity and order in the organization (RIM) at T2 was a moderator for the association between mean HADS at T1 and subsequent bullying (mean NAQ-R at T2). To test H3, I conducted a moderated mediation analysis (model 15 in Hayes’ macro) using SP, calculated as number of days per week at T2, as mediator of the association between mean HADS (T1) and mean NAQ-R (T2), and I used the RIM (T2) as a moderator for both the direct effect from HADS to NAQ-R, and the indirect effect from SP to NAQ-R. In all analyses, sex, age, and the baseline of the dependent variable (workplace bullying) were added as covariates.

## Results

Table 1 presents the means, SDs, and intercorrelations for all variables used in the study. The zero-order correlation between mental health problems at baseline and bullying at follow-up was 0.41. This is the main association that was investigated in the study. RIM was used as a moderator in H2 and H3. Opinions differ regarding the correlation between predictor and moderator. Baron and Kenny (1986), for example, stated that it may be ‘desirable’ (p. 1174) for these to be uncorrelated, while Hayes (2018) argued that the idea that they should be unrelated ‘is a fringe, unorthodox position’ (p. 538). McClelland *et al.* (2017) showed that a correlation between predictors in a moderation analysis is equivalent to the risk of multicollinearity in ordinary regression. I concluded that the observed correlation of *r* = −0.36 between predictor and moderator was low enough to justify continuing with the analyses.

**Table 1.** Means, SDs, and intercorrelations for all study variables (*n* = 921).

	Mean	SD	1.	2.	3.	4.	5.	6.	7.
1. Sex	58% women								
2. Age	50.08	9.76	0.00						
3. Mental health problems, HADS (T1)	0.62	0.46	0.07*	−0.12***					
4. Workplace bullying, NAQ-R (T1)	1.23	0.31	−0.05	−0.13***	0.47***				
5. Workplace bullying, NAQ-R (T2)	1.21	0.31	−0.05	−0.15***	0.41***	0.66***			
6. Self-labelled bullying (T2)	1.06	0.33	0.01	−0.07*	0.16***	0.35***	0.50***		
7. RIM (T2)	5.28	1.22	0.07*	0.13***	−0.36***	−0.34***	−0.43***	−0.22***	
8. SP (T2)	0.09	0.24	0.05	−0.00	0.17***	0.16***	0.23***	0.09**	−0.15***

*P < 0.05.

**P < 0.01.

***P < 0.001.

Testing the first hypothesis, controlling for sex and age, and adjusting for baseline bullying, the OR for the association between baseline mental health problems and bullying at follow-up, for 33 as cut-off for the NAQ-R was 2.41 (95% CI 1.50–3.88), *b* = 0.88, SE = 0.24. When 45 was used as cut-off for the NAQ-R, the OR was 6.19 (95% CI 2.67–14.34), *b* = 1.82, SE = 0.43. For self-labelled bullying reported at least *now and then*, the OR was 3.62 (95% CI 1.77–6.02), *b* = 1.18, SE = 0.31.

**Figure 1.**
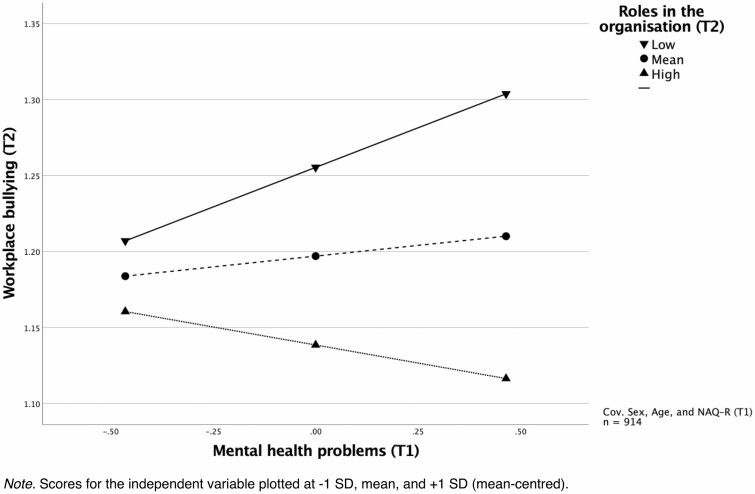
The interaction between roles in the organization and mental health problems with regard to workplace bullying. *Note*. Scores for the independent variable plotted at −1 SD, mean, and +1 SD (mean-centred).

To test second hypothesis, I conducted a moderation analysis controlling for sex and age, and adjusting for bullying at baseline (Table 2). The results showed that role clarity and order in the organization is a moderator, *b* = −0.06, 95% CI [−0.08; −0.04]. Simple slope tests showed that the association between mental health problems and subsequent bullying was only significant when the score on role clarity and order in the organization was low. The slope at 1 SD below the mean is significant, *b* = 0.10, 95% CI [0.06; 0.15]. A Johnson–Neyman test showed that the association becomes significant for values below the 37th percentile on the moderator. For very high values (the 13% highest values) of the moderator, the association becomes positive. The interaction is shown in Fig. 1. I also ran the same analysis predicting self-labelled bullying. The results were similar and the association between mental health and an orderly organization was significant also in this case, *b* = −0.05, 95% CI [−0.08; −0.02].

**Table 2.** Moderation analysis predicting workplace bullying at follow-up (H2).

	*b*	SE *b*	95% CI	*P*
Mental health problems, HADS (T1)	0.03	0.02	[–0.01; 0.06]	0.124
RIM (T2)	–0.05	0.01	[–0.06; –0.04]	<0.001
HADS (T1) × RIM (T2)	–0.06	0.01	[–0.08; –0.04]	<0.001
Workplace bullying, NAQ-R (T1)	0.53	0.03	[0.48; 0.59]	<0.001
Sex	–0.00	0.01	[–0.03; 0.03]	0.818
Age	–0.00	0.00	[–0.00; –0.00]	0.030

*Note.* Dependent variable: NAQ-R (T2). *b* = unstandardized coefficient.

**Figure 2.**
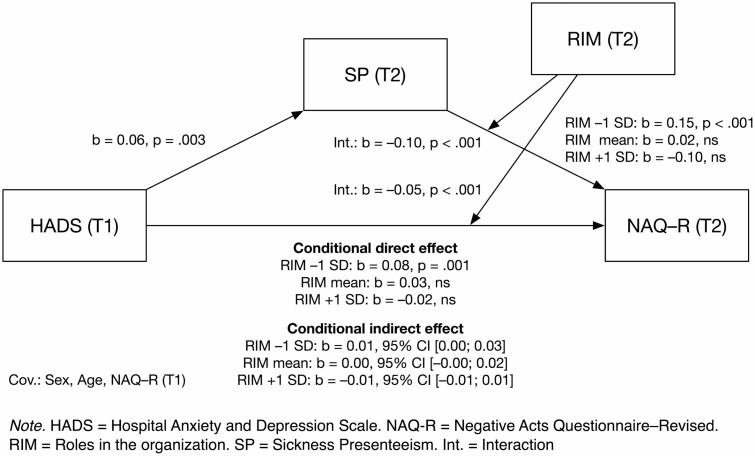
Moderated mediation predicting workplace bullying (H3).

I added SP as a mediator, in order to test the third hypothesis. I tested whether being at work when one should be at home on sick leave affects the association between mental health problems at baseline and bullying at follow-up. A moderated mediation analysis, controlling for sex and age, and adjusting for baseline bullying, showed that SP is a partial mediator. Both the indirect effect and the direct effect depended on the role clarity and order in the organization: for the indirect effect *b* = −0.10, *P* < 0.001, and for the direct effect *b* = −0.05, *P* < 0.001. Fig. 2 shows details of the result. Johnson–Neyman tests showed that the association between baseline mental health problems and subsequent bullying becomes significant for values of the moderator below the 42nd percentile, while the association between SP and bullying becomes significant for values below the 33rd percentile. Low values of the moderator mean low role clarity and order in the organization. For SP, the association with workplace bullying became negative for organizations with very high role clarity and order (above the 94th percentile of the moderator). The index of moderated mediation was significant, −0.01, 95% CI [−0.01; −0.00].

Using self-labelled bullying in the analysis instead of exposure to negative acts showed that SP fully mediates the association between mental health problems and victimization at follow-up. It depends on the role clarity and order in the organization, and SP mediates the association only at low levels (−1 SD): *b* = 0.01, 95% CI [0.00; 0.04]. The index of moderated mediation was significant, −0.01, 95% CI [−0.03; −0.00].

In extended analyses also education, job tenure, immigrant status, and occupational position were added as covariates testing all three hypotheses. These analyses did not change the results presented in the article. Detailed findings from these analyses can be obtained by contacting the author.

## Discussion

The results showed that that mental health problems are associated with an increased risk of being exposed to subsequent bullying, both in the form of exposure to negative behaviours, and victimization (self-labelled bullying), in line with previous findings (see e.g. Nielsen *et al.*, 2014). However, this risk depends on the level of role clarity and order in the organization. The results showed also a partial indirect effect via SP. This means that a person who has mental health problems and comes to work when he or she should have stayed at home runs an increased risk of being bullied. The indirect effect also depends on the level of order in the organization.

The risk that mental health problems will lead to bullying in the current study was almost the same as those found by Kivimaki *et al.* (2003) and by Finne *et al.* (2011). However, the current results show that the risk is only present when an organization fails to provide the basic building blocks of a well-functioning organization—clarity regarding roles and responsibilities, a clear division of tasks, and well-functioning procedures. The same risk factors are suggested by the work environment hypothesis (Leymann, 1996; Einarsen, 2000; Salin and Hoel, 2020) for workplace bullying, regardless of mental health problems. Thus, experiencing mental health problems is not in itself a risk factor, as has been suggested by previous research (see Nielsen *et al.*, 2014)—the current results suggest that employees with mental health problems do not follow special or own rules in terms of the risk for workplace bullying. However, when general risk factors for bullying, such as a poor working environment, are present, those with mental health problems are at greater risk for exposure to workplace bullying.

Research on mental health problems at work has found that cognitive deficits connected to the individual’s tasks, and problems in interpersonal relationships are greater for those with mental health problems (Burton *et al.*, 2004; Neto *et al.*, 2017). It is possible that members of the work group consider that silent agreements about proper conduct at work are being broken, in which case a poor climate in the work group may arise (Zapf *et al.*, 1996). In situations of, for example time pressure or failures, the group may look for a scapegoat or a target for frustration. A person with mental health problems who is already struggling with lack of vigour and a low ability to defend themself—one of the defining aspects of workplace bullying (Einarsen *et al.*, 2020)—may be an easy target. However, the perceived rules and possible violations of them that may act as a trigger for aggression (Felson, 1992) are not left to individual co-workers’ interpretation as much in an orderly organization. In this way, one aspect of the strain–stressor link between mental health problems and bullying is defused, or at least reduced. In a highly well-functioning organization, procedures and strategies for dealing with employees who show symptoms of mental health problems may be in place, as the results indicate. When role clarity and order in the organization is extremely high, the association between mental health problems at baseline and bullying at follow-up is reversed, and the association becomes negative. In a well-functioning organization, the possible negative effects that mental health problems may have for the individual, such as cognitive deficits (Burton *et al.*, 2004; Neto *et al.*, 2017), and negative affect (Aquino and Thau, 2009), seem to be dealt with in a more professional manner, and there may be guidelines for how to best support employees with mental health problems.

On the other hand, in a chaotic organization with poor order, the gloomy perceptions connected to mental health problems suggested by de Lange *et al.* (2005) may be reinforced. If a person persists in going to work, possibly as a tactic to prevent revealing mental health problems, as suggested by McTernan *et al.* (2013), as a consequence of the stigma of mental illness, the reactions and actions of co-workers may be harsh. The results showed an indirect effect of mental health problems through SP to subsequent exposure to bullying. This connection became even clearer as self-labelled bullying, that is, the perception of victimization was fully mediated by SP. If a person with mental health problems endures their work situation, they may feel victimized when exposed to bullying behaviours. An increase in the number of stressors in combination with mental health problems may lead to a vicious circle (Zapf *et al.*, 1996; Nielsen *et al.*, 2014). The perception of victimization may be, at least in part, a result of a higher sensitivity and greater tendency to view behaviours towards oneself as negative, which may lead to a lower threshold for seeing oneself as bullied, as discussed by Nielsen and Einarsen (2018). However, the results of the current study indicate that this explanation only holds true for experiences in organizations that are low in clarity and order.

Interventions aimed at reducing mental health-related absenteeism may in some cases be beneficial for the level of symptoms and for continued employment (Czabala *et al.*, 2011). Previous studies on workplace bullying, however, do not support this, as they portray mental health problems as a risk factor (see e.g. Nielsen *et al.*, 2014). However, the results of the current study support such interventions, but only in well-functioning organizations—mental health problems together with SP increased the risk for workplace bullying only in organizations that were low in order and clarity. To protect employees with mental health problems from becoming victims of bullying, a good start is to make sure that the organization is well functioning with clearly defined roles and responsibilities.

Future research should investigate in more detail the effects that a co-worker with mental health problems may have on the climate in work groups, in terms of the true strain–stressor hypothesis (Zapf *et al.*, 1996). Such a study should look at different organizational conditions, and situations in which different kinds of support are provided. It would also be interesting to study gender differences in a longer perspective, to follow up results that suggest different longitudinal outcomes for men and women (Einarsen and Nielsen, 2015; Rosander *et al.*, 2020). It is important to take the bidirectional relationship between mental health problems and bullying into account, and to investigate other relevant mediators and moderators.

### Strengths and limitations

A strength of this study is that it is based on a probability sample drawn from the total number of employees in Sweden at workplaces with 10 or more employees, and that it uses longitudinal data. The time lag of 18 months and the inclusion of baseline bullying as a covariate are also examples of strengths. As for limitations, all the data are self-reported estimates and the results may be influenced by, for example, common method variance. However, the time lag between the measurements of mental health problems and of workplace bullying should reduce this risk (Podsakoff *et al.*, 2003). Further, the measurement of SP may include presenteeism based on sickness other than a person’s mental health problems, that is, a person with mental health problems may report SP as a result of, for example, a bad cold. However, a certain level of influence from his or her mental health problems is probably part of the estimate and being present at work although one should not––having mental health problems probably would result in the same or similar consequences although the person also had a cold. There were dropouts in the study—a number of people answered only the baseline questionnaire. The attrition analyses showed that the dropouts were people who had been exposed to bullying slightly more than those completing the follow-up. As put forth in the methods section, some of the dropouts may have dropped out because of expulsion from working life, as this risk is higher for victims of bullying (Glambek *et al.*, 2015). In total, the attrition analyses indicate that the result does not overestimate the risks associated with mental health problems, as the dropouts had a greater degree of mental health problems at baseline than those who participated both times.

## Conclusions

In this study, I have investigated *when* mental health problems can lead to workplace bullying. An important finding is that the level of role clarity and order in the organization influences if mental health problems are a risk factor for subsequent bullying. This extends previous research, which has mainly focussed on *whether* mental health problems are associated with bullying. I have also identified a mechanism for *how* mental health problems can lead to bullying. SP partly mediates the association between mental health problems and workplace bullying, that is, if a person persists in going to work, the risk for bullying increases. However, as for the direct effect this risk depends on the clarity and order in the organization. The results extend previous research that has focussed on whether and, if so, why mental health problems lead to SP, and on the consequences for the individual’s work. Workplace bullying does not occur because of the individual, or because of the individual’s deficits: research based on the work environment hypothesis has shown that the main causes of bullying are found in the organization. Individual deficits, such as having mental health problems, are associated with subsequent bullying only when organizational deficits are present.

## Data Availability

The data underlying this article will be shared on reasonable request to the corresponding author.
